# Differentiation Syndrome, a Side Effect From the Therapy of Acute Promyelocytic Leukemia

**DOI:** 10.7759/cureus.12042

**Published:** 2020-12-12

**Authors:** Gizem Reyhanoglu, Benjamin Hughes, Katherine E King, Robert Cambridge

**Affiliations:** 1 Internal Medicine, Lake Erie College of Osteopathic Medicine, Bradenton, USA; 2 Internal Medicine, Nova Southeastern University Dr. Kiran C. Patel College of Osteopathic Medicine, Fort Lauderdale, USA; 3 Anesthesiology, Kansas City University of Medicine and Biosciences, Kansas City, USA; 4 Critical Care Medicine, AdventHealth Orlando, Orlando, USA

**Keywords:** differentiation syndrome, all-trans retinoic acid, atra, acute promyelocytic leukemia, apml, apl, arsenic, ato, pml-rara

## Abstract

Differentiation Syndrome is a complication of all-trans retinoic acid (ATRA) therapy in patients with acute promyelocytic leukemia (APML). It appears clinically as acute end-organ damage with peripheral edema, hypotension, acute renal failure, and interstitial pulmonary infiltrates. When symptoms develop, physicians are recommended to stop ATRA therapy to minimize complications and reduce mortality immediately. This case report describes a 67-year-old male who was diagnosed with acute promyelocytic leukaemia after he developed episodes of hematuria and easy bruising at home. After beginning a treatment regime of ATRA, steroids, and arsenic, the patient began to have symptoms of differentiation syndrome.

## Introduction

Acute promyelocytic leukemia (APML) is primarily due to the expression of the chimeric gene produced through the translocation that occurs between chromosomes 15 and 17 [1]. Promyelocytic leukemia/retinoic acid receptor alpha (PML-RARA) protein results in a blockade in the differentiation of leukemic cells at the promyelocytic stage [1]. Individuals diagnosed with APML are recommended to immediately start all-trans retinoic acid (ATRA), arsenic trioxide, and chemotherapy to prevent the coagulopathy that can be detrimental [2]. Differentiation syndrome (DS), also known as Retinoic Acid Syndrome, is a life-threatening complication that can occur in patients with APML that are undergoing therapy with ATRA or arsenic [3]. This occurs in 2-27% of patients being treated with ATRA or arsenic and usually occurs within a few weeks of initiation of therapy [1]. Although the mechanism of DS is still unclear, it is thought to be due to changes in cytokine secretion and ATRA-induced differentiation of adhesion molecules in APML cells [1]. Diagnostic and peak leukocyte counts, greater than 10 x 10^9/L, as well as abnormal creatinine levels are considered predictive factors for DS [4]. DS is characterized by fever, weight gain of > 5 kg, peripheral edema, hypotension, acute renal failure, and interstitial pulmonary infiltrates [5]. Early recognition of DS, combined with a course of corticosteroid treatment, can significantly decrease morbidity and mortality in these patients [5]. ATRA and arsenic therapy should be stopped immediately when DS is suspected [6].

## Case presentation

Hospital Course and Case Presentation

A 67-year-old male with a past medical history of prostate cancer status-post radiation therapy, hypertension, type II diabetes mellitus, and tobacco use disorder had a recent history of hospitalization due to hematuria and difficulty controlling minor bleeding at home. He noted uncontrolled bleeding after flossing his teeth and from shaving his beard. The patient presented to the hospital with the complaint of having found several “red spots” on his body, which were later noted to be diffuse petechiae. He was initially found to have leukopenia and profound thrombocytopenia.

Upon admission, the hematology/oncology team was consulted. Computed tomography of the chest was ordered to rule out metastases because of the patient’s previous history of prostate cancer. It revealed a focal infiltrate in the posterior right upper lobe and ground-glass nodules in the left upper lobe, along with emphysema. Bone marrow pathology revealed acute myeloid leukemia, highly suspicious for the M3 subtype. Acute promyelocytic leukemia with 80% blasts/promyelocytes, was diagnosed with fluorescent in-situ hybridization (FISH) analysis. He began treatment with ATRA), dexamethasone, and arsenic trioxide (ATO) on 08/04/2020 (Table 1).

**Table 1:** Lab Values Before and After Administration of APML Therapy APML- Acute promyelocytic leukemia; BUN- Blood urea nitrogen; WBC- White Blood Cells

Date	Creatinine (range: 0.84- 1.2 mg/dL)	BUN (range: 7 to 20 mg/dL)	WBC Count (4.5- 11 x 10^9/L)
8/4/20	1.92	64	42.91
8/5/20	3.19	103	28.67
8/6/20	4.26	111	31.53
8/7/20	5	117	10.18
8/8/20	5.26	123	3.56
8/9/20	5.16	108	1.16
8/10/20	2.73	70	1.2
8/11/20	2.79	75	3.18
8/12/20	3.85	104	2.92
8/13/20	3.8	98	3.56
8/14/20	3.53	95	2.59
8/15/20	2.73	72	1.55
8/16/20	2.44	64	2.03

**Figure 1:**
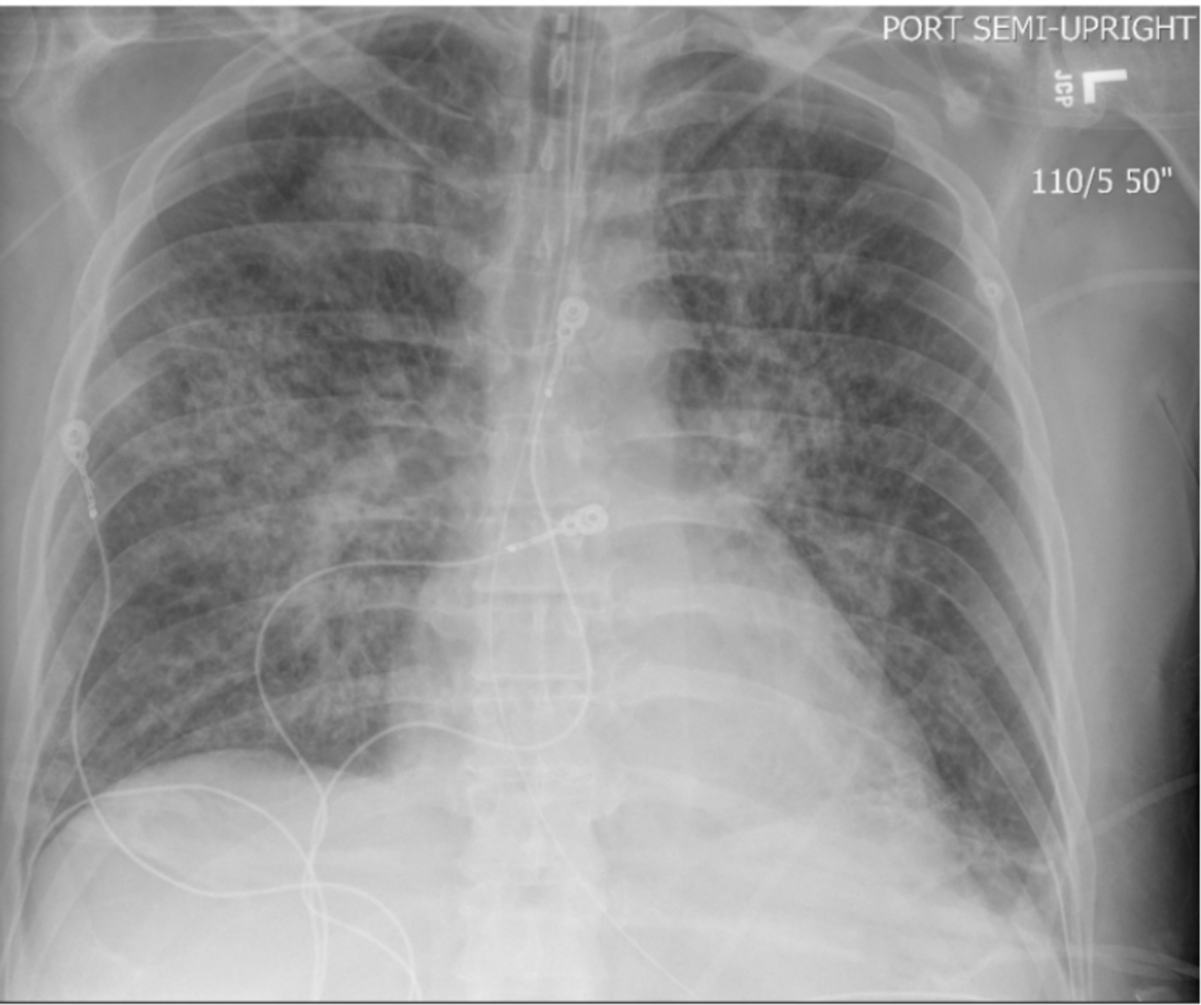
Chest X-Ray 08/05/2020

**Figure 2:**
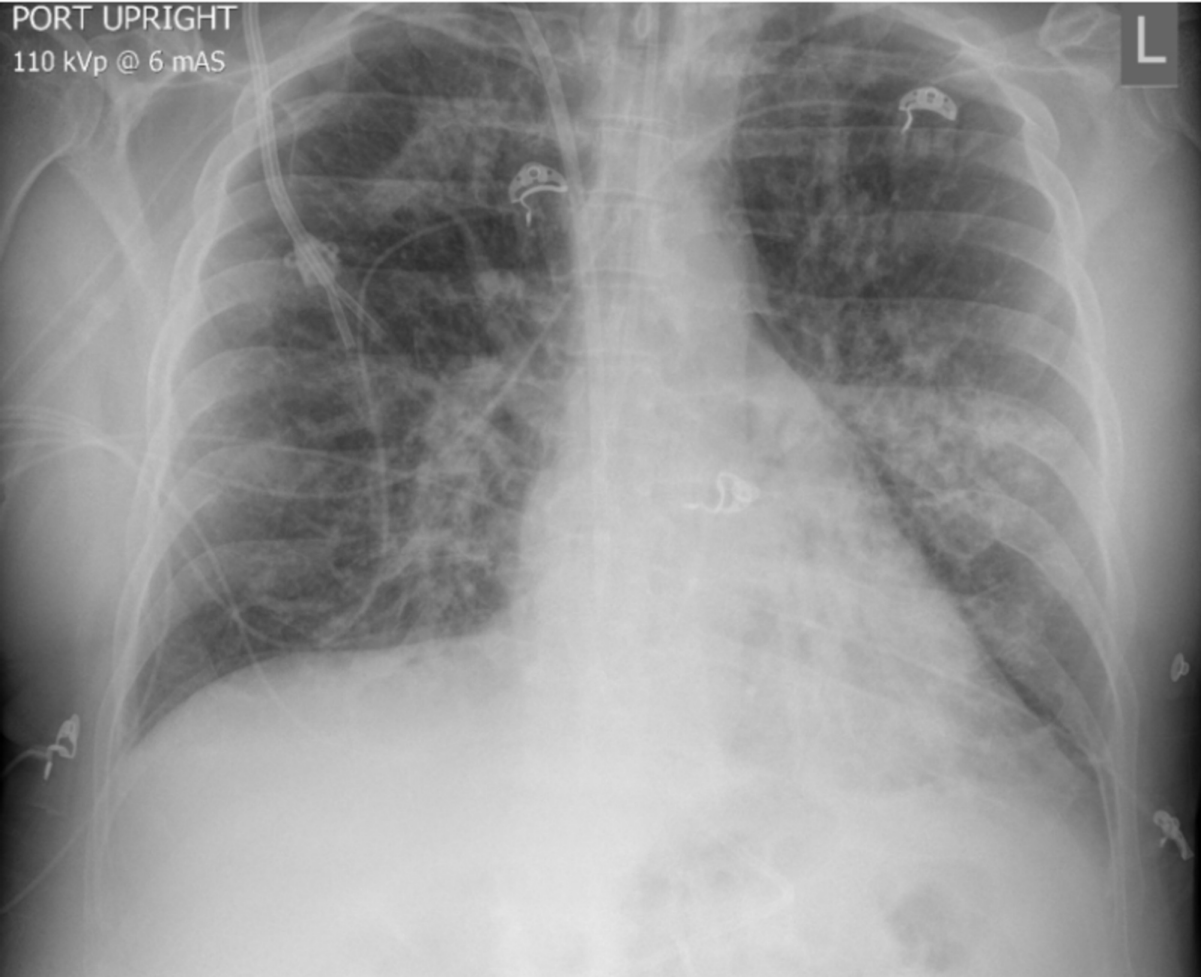
Chest X-Ray 08/19/2020

After starting ATRA, dexamethasone, and ATO therapy, the patient complained of chest tightness and dyspnea, for which he was given furosemide. The patient’s liver enzymes and white cell count began to elevate rapidly over the next several days (Table 1). He also developed atrial fibrillation with a rapid ventricular response and was placed on metoprolol. Continuing investigation revealed further white cell count elevation, decreased oxygen saturation, acute kidney injury, and a chest x-ray that showed increased infiltrations and worsening pulmonary edema (Figure 1 and 2). The patient was then transferred to the intensive care unit for worsening hypoxemic respiratory failure, hypotension, and acute renal failure (Table 1). He ultimately required intubation, vasopressor support, and initiation of continuous renal replacement therapy (CRRT). ATRA and ATO were held due to concern for worsening symptoms secondary to suspected differentiation syndrome (DS). The patient was then started on one dose of chemotherapy: cytarabine, for debulking and restarted on ATRA two days later, as literature does not recommend holding therapy for APML for too long.

After the initial signs of DS had resolved, the oncology service and critical care service tried to balance the need for ongoing chemotherapy against his current clinical condition. ATO was held until labs revealed improvement of liver enzymes. The patient continued ATRA and dexamethasone and was given a single dose of idarubicin. The patient was finally extubated 11 days after the initial stop of his therapeutic regimen. CRRT was stopped on the same day, but renal function had not significantly improved, so he was converted to conventional hemodialysis. The patient has been stable since the restart of all medications, waiting for a bone marrow transplant in the hospital.

## Discussion

ATRA is associated with cellular migration, endothelial activation, and tissue damage from interleukins, leading to DS [7]. The all-trans retinoic acid and idarubicin (AIDA) regimen is used for patients who are recently diagnosed with APML. Both of these medications had been initiated at different periods in this patient’s course. About one-quarter of patients with APML, who undergo induction therapy with the AIDA regimen, will develop DS [3]. The development of DS often presents with an elevated white blood cell count and occurs between 7 to 12 days following induction therapy [3]. The patient, in this case, presented with a much more rapid onset of DS following induction therapy. The most frequent symptoms associated with DS include dyspnea, pulmonary infiltrates, unexplained fever, pleural effusion, and renal failure; all of which were noted to occur with the patient after induction therapy [3].

Management for DS has remained more-or-less unchanged throughout the years. Prophylactic corticosteroids are generally used in patients with APML on therapy with ATRA to prevent DS [2]. Although their benefit is not fully understood, it can still be considered. It is supported by the LPA99 trial, which provided candidates with prophylactic prednisone from the initiation of ATRA for 15 days [3]. Corticosteroids, such as prednisone prophylaxis, do not reduce mortality from DS but do reduce the incidence of severe DS. It is recommended that physicians avoid drugs that have been known to cause QT prolongation, as arsenic trioxide can also cause QT prolongation [2]. The patient in this case report had to stop his ATO due to this side effect. There should be temporary discontinuation of ATRA or ATO, as seen in this case report until end-organ damage shows improvement [2]. Abnormal serum creatinine can be considered an independent predictive factor for severe DS [3].

## Conclusions

The patient, in this case, was started on conventional hemodialysis three times a week. His recovery was slow, but he did continue his regimen for APML once he showed signs of stabilization. Other than trending end-organ damage markers to understand the progression of differentiation syndrome, physicians need to look for the symptoms associated with DS before it is too late. Using prophylactic corticosteroids, as seen in this patient, does not pose any harm at this point, but further research into their mechanism of action could be crucial in assisting future APML patients who have an adverse event while on ATRA.
